# Biomimetic Models to Investigate Membrane Biophysics Affecting Lipid–Protein Interaction

**DOI:** 10.3389/fbioe.2020.00270

**Published:** 2020-04-15

**Authors:** Joe Sarkis, Véronique Vié

**Affiliations:** ^1^Department of Cell Biology, Harvard Medical School and Program in Cellular and Molecular Medicine, Boston Children’s Hospital, Boston, MA, United States; ^2^Univ Rennes, CNRS, IPR-UMR 6251, Rennes, France

**Keywords:** cell membrane, lipid–protein interactions, bioengineering, *in vitro* models, membrane biophysics, binding kinetics

## Abstract

Biological membranes are highly dynamic in their ability to orchestrate vital mechanisms including cellular protection, organelle compartmentalization, cellular biomechanics, nutrient transport, molecular/enzymatic recognition, and membrane fusion. Controlling lipid composition of different membranes allows cells to regulate their membrane characteristics, thus modifying their physical properties to permit specific protein interactions and drive structural function (membrane deformation facilitates vesicle budding and fusion) and signal transduction. Yet, how lipids control protein structure and function is still poorly understood and needs systematic investigation. In this review, we explore different *in vitro* membrane models and summarize our current understanding of the interplay between membrane biophysical properties and lipid–protein interaction, taken as example few proteins involved in muscular activity (dystrophin), digestion and Legionella pneumophila effector protein DrrA. The monolayer model with its movable barriers aims to mimic any membrane deformation while surface pressure modulation imitates lipid packing and membrane curvature changes. It is frequently used to investigate peripheral protein binding to the lipid headgroups. Examples of how lipid lateral pressure modifies protein interaction and organization within the membrane are presented using various biophysical techniques. Interestingly, the shear elasticity and surface viscosity of the monolayer will increase upon specific protein(s) binding, supporting the importance of such mechanical link for membrane stability. The lipid bilayer models such as vesicles are not only used to investigate direct protein binding based on the lipid nature, but more importantly to assess how local membrane curvature (vesicles with different size) influence the binding properties of a protein. Also, supported lipid bilayer model has been used widely to characterize diffusion law of lipids within the bilayer and/or protein/biomolecule binding and diffusion on the membrane. These membrane models continue to elucidate important advances regarding the dynamic properties harmonizing lipid–protein interaction.

## Introduction

Biological membranes are fundamental elements for cellular organization. They provide cellular entities and are responsible for the compartmentalization of cytoplasmic space into functionally specialized organelles as well as controlled exchanges between the interior of the cell and the extracellular environment. These membranes are far from being inert envelopes. The “fluid mosaic” model was introduced by Singer and Nicolson (1972), analogous to a two-dimensional oriented solution of integral proteins (or lipoproteins) in the viscous phospholipid bilayer solvent. Biological membranes described as consisting of a double layer of phospholipids, in which the hydrophobic chains face each other, traversed by membrane proteins. The lipids are in a perpetual movement of lateral diffusion, via the Brownian motion (Lipowsky and Sackmann, 1995), and the membrane proteins also move, but more slowly than the lipids which surround them. With this enormous complexity and huge diversity of lipids; not only between species but also in different membranes in one cell; it is clear that in order to understand the key parameters in the lipid–protein interactions, we are tempted to simplify our experimental conditions, thus the appearance of basic membrane models. Using such reductionist approach, it is possible to retrieve information about the physical rules which regulate the phase behavior of the membranes and the interplay between protein and lipids. The most well-known and common biomimetic system used for such purposes are discussed here: lipid monolayer, lipid vesicles, and supported lipid bilayers (SLB) with a brief example of bicelles. While each of these systems exhibits advantages and disadvantages, it is clear that the exploitation of various model systems and different investigation techniques offers a better understanding of the complex lipid/protein interactions which might be relevant to accomplish membrane functions. Note that proteins that are used here, mainly dystrophin and DrrA, are discussed in depth only to emphasize different examples where lipid biophysical properties modulate lipid–protein interaction.

## Lipid Monolayer Models

The understanding of monolayer formation at the liquid/air interface has been forged over the last few centuries by the experiments carried out by B. Franklin in the 18th century (1774), followed by Rayleigh, Pockels, and Gibbs in the 19th century (Rayleigh, 1890), but it is I. Langmuir (Langmuir, 1917) Nobel Prize winner in 1932 who developed the technique to control the formation of monolayers and their thermodynamic studies. Together with K. Blodgett, they develop a technique to transfer monomolecular films to a solid support in order to expand analytical techniques (Blodgett, 1935).

The interest of biologists in monolayers appeared after a remarkable study by Gorter and Grendel (1925) who based the hypothesis of the structure of biological membranes as the combination of two monolayers after spreading a chromocyte lipid extract on a Langmuir trough. Thus, the spontaneous formation of lipid monomolecular film at the interface makes it possible to mimic one of the leaflets of the biological membrane. The choice of lipids will be guided by the type of leaflet to be studied. Thus, a mixture of phosphatidylcholine (PC), sphingomyelin (SM), cholesterol (CHOL), will be representative of the outer leaflet of the biological membrane. The replacement of sphingomyelin by an anionic lipid as phosphatidylserine (PS) or phosphatidylglycerol (PG) will represent the inner leaflet (Devaux and Zachowski, 1994). To mimic the bacteria membranes, the major component of the plasmic eukaryotic membrane phosphaditylcholine is usually replaced by phosphatidylethanolamine (PE) and the use of extracted lipopolysaccharides from bacteria will represent the external membrane in antimicrobial activity studies (Lugtenberg and Peters, 1976; Epand et al., 2007; Legrand et al., 2011; Derde et al., 2015a, b). Recently, the molecular mechanism of amphotericin B antimicrobial activity was characterized using monolayer (Wang et al., 2020) and showed the importance of amphotericin B interaction with sterol by increasing monolayer fluidity. Another exciting usage of lipid monolayer models is to investigate lung surfactant monolayer and how it interacts with drugs related to lung diseases (Hu et al., 2019). When inhaling Ketoprofen, the molecule hinders the formation of liquid-condensed films and affect the property of the membrane during breathing. In a different lung model study, two air pollutants (Stachowicz-Kuśnierz et al., 2020) was shown to adsorb and accumulate in the hydrophobic part of the monolayer.

### Langmuir Trough

Langmuir trough equipped with mobile barriers has been widely used to study the thermodynamic behavior and 2D phase diagrams of purified lipids or in binary mixtures (Vié et al., 2000). Ternary mixtures have also been examined including the effect of cholesterol which is an essential element of biological membranes by modifying rheological properties (Brown and Brockman, 2007; Guyomarc’h et al., 2014).

In this part of the review, Langmuir trough will be presented in the context of protein/membrane interactions while manipulating surface pressure measured through the Wilhelmy plate by controlling barriers movements. Here, we will not discuss the interactions that lead to lipid degradation as in the context of enzymatic activity (Verger and De Haas, 1973). In these studies, the authors often use a zero order trough, which consists of two compartments connected by a channel that maintains both surface pressure and area constant during the degradation and solubilization of reaction products (Sias et al., 2004).

Due to the structure of the interface, the proteins studied are essentially part of peripheral proteins in the domain of animal or vegetal (Bottier et al., 2008; Chièze et al., 2011; Sarkis et al., 2014) or proteins with membranotropic activity such as antimicrobial activity (Legrand et al., 2011; Derde et al., 2015a, b). The Langmuir trough is associated with surface pressure measurement system but over the years this technique has been associated with many other techniques, notably based on the properties of the reflection of light (ellipsometry, Brewster angle microscopy, infrared spectroscopy) or neutron and X ray (Berge and Renault, 1993; Estrela-Lopis et al., 2001; Miller et al., 2005; Bello et al., 2016).

It is through two examples, a study of the interactions between dystrophin-lipids and an investigation of mechanism of gastric milk fat globule digestion that we will describe the use of the methods developed around the Langmuir trough to highlight the different types of interaction.

### Impact of the Lateral Lipid Pressure on Lipid–Protein Interactions

The power of lipid monolayer relays on understanding the physico-chemical properties of biomembrane, where packing and compressibility of the lipid could be controlled by monitoring the surface per area per lipid molecule. Recently, Pinto and Disalvo (2019) proposed a novel monolayer model as an open system based on thermodynamics of irreversible process. Some new investigations listed here will emphasize the usage of such model to characterize molecular mechanisms and the importance of lateral lipid pressure after protein binding. The impact of ions (such as calcium) on lipid–protein interactions was crucial in stabilizing the complex formation at the membrane as shown by Krajewska et al. (2020). Moreover, Shiga toxin binding to its cellular receptor (glycolipid) will initiate narrow membrane bending due to lipid compression and long-range membrane reorganization (Watkins et al., 2019).

**FIGURE 1:**
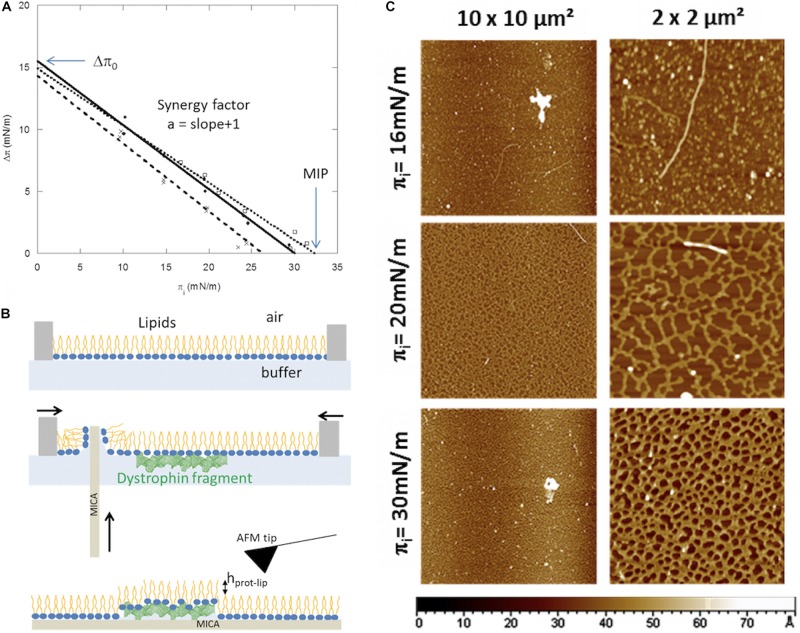
**(A)** Insertion of dystrophin fragments, R4-9 (?) R11-15 (∙) R16-21 (x), increases surface pressure of lipid monolayer and is modulated by the lipid initial surface pressure (π_i_). The maximal insertion pressure (MIP) and the theoretical pressure increase at the lipid-free interface (Δπ_0_) are indicated by arrows. Adapted and modified from Sarkis et al. (2011) and Ameziane-Le Hir et al. (2014). **(B)** Schematic representation of the Langmuir-Blodgett transfer of the interfacial protein/lipid film on solid support (such as mica). Scale of protein and lipid size in this illustration is not respected. Adapted from Vié et al. (2018). **(C)** AFM images of the R11-15/DOPC:DOPS monolayers transferred at the end of the protein adsorption on lipid monolayer at different initial surface pressure, 16 mN/m, 20 mN/m, and 30 mN/m. Adapted from Sarkis (2012).

Adsorption experiments at lipid/liquid fluid interfaces are used to characterize protein–lipid interactions. Lipids are spread to the liquid surface using a high precision syringe. The lipids are usually diluted in chloroform/methanol. The lipids are compressed at the desired surface pressure, initial surface pressure (π_i_). Protein is then injected in the subphase at the chosen concentration. Biological membrane are in a fluid state and its inner leaflet carried out negative charges, therefore, 1:1 molar ratio of dioleoylphosphatidylcholine (DOPC) and dioleoylphosphatidylserine (DOPS) are chosen (Fiehn et al., 1971; Devaux and Zachowski, 1994). The presence of unsaturation on the hydrocarbon chains allows remaining in the expanded liquid state whatever the lateral pressure. The surface pressure is directly related to the lateral cohesion of molecules and initial interfacial pressure values are related to the lateral packing of the lipids before protein interactions. Low surface pressure will promote insertion of amphiphilic proteins through access to the hydrophobic parts of the lipids, so electrostatic and hydrophobic forces can be brought into play in the surface pressure range between 10 and 20 mN/m. When lateral pressure increases, the polar head-groups come close together and as a result the access of hydrophobic parts decreases, limiting the protein insertion. Electrostatic interactions can persist, and proteins will mainly interact with polar head-groups. The graph in Figure 1A shows the surface pressure variation (Δπ) induced by protein/lipid interaction versus the initial pressure (π_i_) of the monolayer for three purified fragments of the central dystrophin domain, DYS R4-9, DYS R11-15, DYS R16-21 (Sarkis et al., 2011; Ameziane-Le Hir et al., 2014). Indeed, the dystrophin is the most important muscular protein essential for maintaining the integrity of the muscular cell during elongation/contraction cycles (Koenig et al., 1988). We studied possible interactions between this central domain constituted by 24 spectrin-like repeats noted R and the plasma membrane. As expected, the insertion decreases as the initial surface pressure increases. Curve analysis of Δπ values *vs.* π_i_ will identify three parameters related to the binding of each fragment as described by Calvez et al. (2011) and Boisselier et al. (2012): Δπ_0_, synergy factor, and maximal insertion pressure (MIP). Δπ_0_, value determined by extrapolation for π_i_ equal to 0, is the theoretical pressure increase at the lipid-free interface (π_initial_ = 0 mN/m). The synergy factor (a) is obtained by addition of 1 to the slope of the linear regression and is related to the protein affinity for the lipid monolayer. MIP is the curve extrapolation intercept with x-axis; it identifies the lipid monolayer initial surface pressure for which the dystrophin fragment injection does not induce increase in surface pressure.

Linear regression for the three fragments (*R*^2^ comprises between 0.98 and 0.99), and the parameters deduced from these equations are presented in Table 1. Surface pressures measured at the lipid free interface (p_e_) are also reported for these three fragments. For all samples, Δπ_0_ are around 15 mN/m, DYS R16-21 presents a lower value than DYS R11-15 and DYS R4-9. Values are smaller than the adsorption of these dystrophin fragments at the lipid free interface (air/liquid) at the same subphase concentration (Table 1) indicating the presence of lipid limits the interface accessibility of the protein, probably by steric hindrance.

**TABLE 1:** Interaction of protein with monolayer at different surface pressure: summary of the values of Δπ_0_, synergy factor and MPI of 3 different fragments of dystrophin.

		R4-9	R11-15	R16-21
**Lipid/liquid interface**	Linear regression	*y* = −0.51x + 15.47	*y* = −0.46x + 14.9	*y* = −0.54x + 14.29
	Theoretical Δπ_0_ (mN/m)	15.47	14.9	14.29
	Synergy factor	0.48	0.54	0.45
	MIP (mN/m)	30.0	32.3	26.3
**Air/liquid interface**	π_e_ (mN/m)	19.1	20.4	21

Nevertheless, when values are positive, the synergy factor shows the presence of attractive interactions between lipid and protein, in addition of the protein surface activity, since its insertion into the lipid occurs even at initial surface pressure higher than Δπ_0_ or than p_e_. Values close to 0.5 are the range of values reported in the literature for strong protein/phospholipid interactions with cumulative effect of the electrostatic and hydrophobic forces.

The MIP discriminates in the same way the three fragments. DYS R11-15 and DYS R4-9 have values higher than DYS R16-21. Moreover, these values are greater than the surface pressure supposed of natural membrane systems in eukaryotic cells which is around 30 mN/m (Marsh, 1996). These experiments highlight differences of protein behavior against a lipid monolayer in the dystrophin central domain. The less efficient binding of R16-21/lipids could be attributed to the hinge location between R19 and R20 reducing the anchorage of this fragment in the membrane.

Atomic force microscopy (AFM) visualization of monolayers provides complementary information about the molecular protein organization in lipid films, and how this organization is affected by the initial surface pressure. For imaging at the molecular resolution, the monolayer was transferred using the Langmuir-Blodgett method described in Figure 1B. Usually, and especially for biological samples, the mica substrate is selected because its atomic flat surface does not affect the topography. The color scale on the images is related to the height variations. Thanks to the size differences between lipids and proteins, the protrusions are credited to the existence of the proteins or lipid-protein complexes. DYS R11-15 organization in DOPC:DOPS monolayers has been described depending on the lipid initial surface pressure (Sarkis et al., 2011). As observed in the images presented in Figure 1C, at low π_i_ (16 mN/m), the proteins are dispersed as diluted in the lipid film while the high π_i_ (20 mN/m and 30 mN/m) favor the protein/protein interactions to form a homogeneous network. The protein network height increases with the initial surface pressure, from 1.1 nm to 1.8 nm, showing that even though the protein insertion is lower at 30 mN/m, the proteins are stabilized beneath the interface by interactions with the lipid headgroups.

**FIGURE 2:**
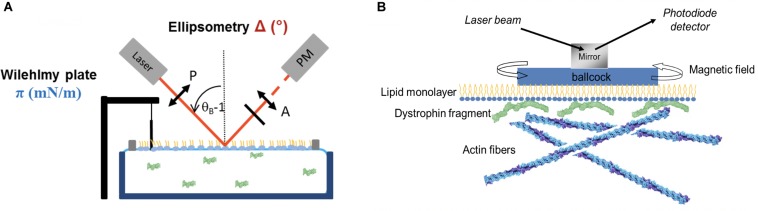
**(A)** Illustration of Langmuir trough associated with Wilhelmy plate and the ellipsometry setup. Adapted from Vié et al. (2018). **(B)** Interfacial rheological setup used to measure lipid monolayer rheological properties when interacting with dystrophin fragment and actin filaments. Adapted from Sarkis (2012).

To test the presence of molecules interacting essentially with the headgroup when the surface pressure is slightly affected or even decreases, a very useful tool is the ellipsometry in “null ellipsometer” configuration which gives the ellipsometric angle noted D (°) (Berge and Renault, 1993). The polarized beam probes an area of 1 mm^2^ with 1 μm depth. D is relative to both the reflectivity index and the thickness of the film (Figure 2A). In association with AFM imaging, the value of D can discriminate between different interfacial mechanisms: none protein/lipid interaction, a lipid reorganization inducing a surface pressure decrease or the protein accumulation under the lipid films. A study focused on the interaction between a gastric lipase (recombinant dog gastric lipase, rDGL) and lipid monolayers mimicking the surface of milk fat globules was described in Bourlieu et al. (2016). Different lipid mixtures were used to highlight the impact of the lipid nature on the rDGL adsorption. For the same initial surface pressure, the lipid packing was modulated changing the head-group nature as phosphatidylethanolamine (PE), phosphatidylcholine (PC) and phosphatidylserine (PS), this latter lipid being anionic. In addition, the mixture of saturated (DPPC) and unsaturated (DOPC) lipids leads to phase coexistence called liquid expanded (DOPC) and liquid condensed (DPPC) at 20 mN/m. Thus, the mechanisms of rDGL adsorption had been described, first a preferential insertion into the expanded liquid phase allowing the protein anchorage; second in all lipid mixture the values of D increased from 3 to 18° and the surface pressure variation between 0 and 5.4 mN/m, indicating a strong accumulation of the protein under the film. In addition, the higher values of D and p variations were obtained in presence of anionic lipids suggesting that the electrostatic forces are involved in the adsorption process. As conclusion, the large number of lipase enzyme located just below the lipid interface is related to the physiological digestive process favoring a rapid fat hydrolysis of the milk throughout neonatal digestion. Moreover, it was shown that the lipid phase impacts the insertion of rDGL. Indeed, comparison between lipid membrane of fat milk globules of human and bovine highlights the positive effect of the presence of polyunsaturated lipid chains on the lipase stabilization and organization in the lipid interface resulting of an enhanced lateral compressibility (Bourlieu et al., 2020).

### Membrane Rheology and Lipid–Protein Interaction

The lipid monolayer is studied to understand the possible mechanism of protein anchorage in the membrane. Nevertheless, in some cases, proteins can interact simultaneously with other cytoplasmic structures forming a bridge or linker with the membrane. To demonstrate the presence of these multiple interactions, the shear elastic constant and surface viscosity can be measured using home-made interfacial rheometer (Figure 2B) (Vénien-Bryan et al., 1998). Different techniques to investigate the interface of lipid monolayer or protein films were described in the literature (Walder et al., 2008; Krägel and Derkatch, 2010; Le Floch-Fouéré et al., 2010).

The dystrophin fragment DYS R11-15 interacts strongly with the membrane as describe above, nevertheless, this fragment was described to bind actin filament (F-actin) (Rybakova, 1996; Amann et al., 1998). So, the two questions came to mind: is it possible for DYS R11-15 to establish interaction simultaneously with the lipid monolayer and the F-actin? And how rheological properties could be affected? For this purpose, we measured the shear elastic constant and the viscosity using the setup presented in the Figure 2B. Two Helmoltz coils are used to apply a torque on the magnet placed in a float on the lipid monolayer interface. The amplitude of rotation of the float depends of the rheological properties of the monolayer. We applied the model of Kelvin-Voight solid to calculate shear elastic constant (μ, mN/m) and surface viscosity (η, N⋅s/m). Different conditions were tested: two lipid mixtures (DOPC:DOPS and DOPC:DOPE), three subphase DYS R11-15 concentrations, injection of G-actin versus F-actin (Sarkis et al., 2013). For the DOPC:DOPS mixture, the μ values increased slightly with the lipid initial surface pressure between 20 mN/m and 27 mN/m(from 3 to 4 mN/m) and decreased to zero at 30 mN/m (close to the MIP) in accordance with the amount of protein inserted. DYS R11-15 doesn’t interact with G actin but addition of ATP + Mg^2+^ inducing actin polymerization triggers a significant increase of the elastic constant and surface viscosity.

These studies showing that DYS R11-15 plays the role of a linker between the membrane and the F-actin bring new insight on the essential function of the dystrophin in maintaining the integrity of muscle fiber membranes during contraction/elongation cycle.

Experiments on Langmuir trough mimicking one membrane leaflet seems to be too simplistic model relative to the complexity of a biological membrane, whereas the second leaflet is neglected, and the proteins studied are limited to the peripheral proteins. Nevertheless, by controlling the physico-chemical parameters, it is possible to understand interactions, identify and quantify them and elaborate scenarios of processes involved *in vivo*.

## Lipid Bilayer Models

Biomimetic systems ease the interpretation of the measurements that generally prove challenging with the complex composition of *in vivo* membranes. Though simplification is the goal, building these models still requires multiple steps. The most used membrane bilayer models are lipid vesicles such as small unilamellar vesicles (SUV also known as liposomes with a size range of 30–50 nm), large unilamellar vesicles (LUV with a size range 100–500 nm) and giant unilamellar vesicles (GUV with a size higher than 1 μm) as well as SLB on a solid substrate.

There are also other membrane models called bicelle or nanodisc. The mixture of long-chain and short-chain lipid or amphiphiles or proteins allows self-assembled discoidal lipid bilayers called bicelle (Lin et al., 1991; Sanders and Landis, 1995; Van Dam et al., 2004; Dürr et al., 2013; Dos Santos Morais et al., 2017) or nanodics (Bayburt et al., 2002; Schuler et al., 2012; Rouck et al., 2017). The morphology and size are very sensible to the ratio long/short chain, called q, temperature and dilution as described in the literature (Raffard et al., 2000; Glover et al., 2001; Harroun et al., 2005). Widely used in NMR (solid and liquid) thanks to their ability to align in a magnetic field, they have made it possible to obtain the structure of membrane proteins in their lipid environment, such as that of cytochrome b5, or that of a protein of the HIV virus envelope (Dürr et al., 2007; Dev et al., 2016). While the membrane proteins are embedded in the bilayer (flat part of the bicelle), structural information can be obtained on peripheral proteins when it interacts either with the flat part (Loudet et al., 2005) or with the curved part (Dos Santos Morais et al., 2018). When investigating the bicelle to vesicle transition using coarse-grained molecular dynamics simulations, the local lipid composition is the key factor determining the success of vesicle formation (Koshiyama et al., 2019). Nanodics were used to solve mechanism of membrane fusion mediate by membrane fusion protein (Shi et al., 2013) or of Ca^2+^ transport through membrane protein (Conrard and Tyteca, 2019). Such systems are mainly dedicated to the structural protein or peptide studies, therefore, we focused in this review on vesicles and supported bilayers.

### Lipid Vesicles and Curvature Effect on Protein Binding

Vesicle formation starts with combining a mixture of lipids in chloroform/methanol followed by a dehydration step under vacuum or other inert gas such as nitrogen or argon to remove the bulk organic solvent, avoiding oxidation and a lipid film is formed on the surface of the glass tube. Resuspension of the dried film in buffer generate a multilamellar vesicles (MLV) solution.

SUVs are obtained by sonication of an MLV suspension or by filtration through a very small filter. The strong curvature imposed on the membrane implies an external surface significantly greater than the surface of the inner monolayer and therefore a higher number of lipids in this outer monolayer. The small size of SUVs favors their use in optical spectroscopy.

LUVs are obtained by extruding an MLV solution through filters with calibrated pore sizes. LUVs, like SUVs, can be produced in a saline medium and in particular physiological. Phase reversal evaporation is another technique for preparing LUVs (Rigaud et al., 1983). This method employs forcing the emulsion by sonication between an ether phase (lipid solvent) and an aqueous phase. The ether is then removed slowly by evaporation under partial vacuum. The curvature of a large vesicle is closer to physiological reality. These are much more diffuse objects than SUVs.

GUVs have a size ranging from 1 μm to more than 100 μm in diameter. They are formed in a non-ionic medium by electroformation (Angelova and Dimitrov, 1986) or by gentle hydration in an ionic medium (rehydration of lipids with or without an electric field). A very good amount of unilamellar vesicles are obtained by electroformation (80%), whereas a poor yield of unilamellar vesicles is obtained by gentle hydration. Only the formation of GUVs by gentle hydration can be performed in a physiological medium (Rodriguez et al., 2005). Other techniques for the preparation of GUV exist, specifically by double emulsion: where a drop of aqueous solution passes successively through two interfaces between an oil containing the lipid and an aqueous solution (Stachowiak et al., 2008). GUVs are mostly used to decrypt some membrane mechanisms such as diffusion of membrane proteins, membrane deformations, lipid raft formation, phase segregation.

The size and monodispersity of the vesicles are verified by electron microscopy or light scattering. From a general point of view, the vesicles (MLV, SUV, and LUVs) can be produced at high concentrations of the order of 10 mg/mL and are very resistant.

When they form vesicles, the lipid bilayers limit the exchanges between the internal compartments and the outside. Pure bilayers are impervious to ions and large polar molecules, and only partially permeable to water that can intrude between lipids under the effect of osmotic pressure. This means that the physiological functions of biological membranes cannot simply rest on the bilayer but require the presence of other components such as membrane proteins to allow controlled exchanges between the intracellular and extracellular environments.

**FIGURE 3:**
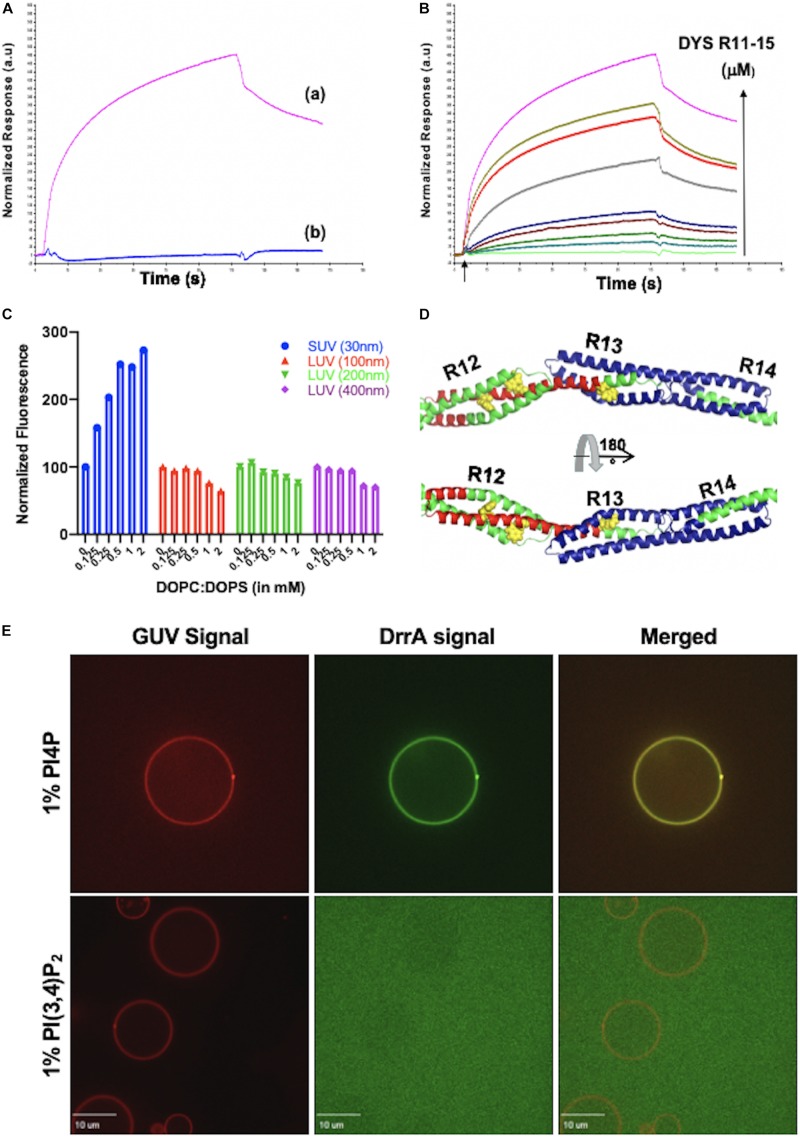
**(A)** Surface Plasmon Resonance traces showing the binding of DYS R11-15 to DOPC:DOPS **(a)** and to DOPC:DOPE **(b)** SUVs. **(B)** SPR traces of DYS R11-15 binding to DOPC:DOPS SUVs in a dose dependent manner. **(A,B)** Figures are adapted from Sarkis (2012). **(C)** Fluorescence intensity of tryptophan residues in DYS R11–15 (0.5 μM) after 2 h of incubation with different concentrations of DOPC/DOPS. SUV diameter was 30 nm and LUV of three different sizes: 100, 200, and 400 nm. Modified from Sarkis et al. (2011). **(D)** Structure of DYS R12-14 (PyMol) obtained by sequence homology illustrating the protected zone from trypsin in red, due to the direct binding to anionic SUV, the unprotected zone in shown in blue, which is more sensitive to trypsin digestion when vesicles are present. Residues in yellow correspond to Tryptophan. Adapted from Sarkis et al. (2011). **(E)** Confocal microscopy images of different composition of GUV incubated with DrrA. GUVs contains 20% DOPC, 15% DOPS, 35% DOPE, 30% cholesterol and 1% PI4P or PI(3,4)P2. Reproduced by J. S. from Schoebel et al. (2010); Del Campo et al. (2014), and Sidm and Wang (2016).

The interactions of DYS R11-15 with SUVs (anionic and zwitterionic) (Sarkis, 2012) was investigated using surface plasmon resonance (SPR). The first step was to fix liposomes on the sensor chip. Streptavidin was previously immobilized, and SUVs containing 0.1% biotinylated lipids were fixed on the surface by the strong streptavidin-biotin interaction. Then, the injection of DYS R11-15 in the microfluidic system makes it possible to characterize the behavior of the protein binding to the SUV as a function of time. When anionic SUVs (DOPC: DOPS) are attached to the sensor chip, injection of DYS R11-15 (5 μM) increases the signal significantly (from 0 to 300 RU) showing that a protein-lipid interaction is formed (Figure 3Aa). The more DYS R11-15 is injected at a higher concentration, the greater the intensity of the response (Figure 3B). On the other hand, the passage of DYS R11-15 (5 μM) on zwitterionic SUVs (DOPC: DOPE) does not cause any significant signal modification (Figure 3Ab). Under the same experimental conditions, DYS R20-24 generates no signal; this protein has so far been considered as non-interacting with SUVs (Legardinier et al., 2008). This highlights the lipid binding specificity we observed with DYS R11-15.

Vesicle and tubule budding allows different cell compartments to communicate with each other (Heuser and Evans, 1980; Sciaky et al., 1997; Bright et al., 2005). Lipid molecules are able to diffuse freely in each of the bilayer leaflets thanks to the fluidity of the cellular membranes (Singer and Nicolson, 1972). The choice of the model system, and mainly the size of the vesicles plays a determining role to characterize protein-membrane interactions. By varying vesicle size, the curvature radius is modified. High curvature can be found in many small vesicles with a radius of 25 nm carrying proteins as well as in organelles with complex structure such as endoplasmic reticulum (Shibata et al., 2009). Lipid composition dictate bio-mechanical properties impacting organelle shapes and modulating their deformation (Antonny, 2011) and the actual shape of cellular membranes thought to largely depends on proteins. An interesting paper using dissipative particle dynamic simulations explored how nanoparticles can adsorb and intake in a lipid bilayer (Burgess et al., 2020). Much progress has been made to understand these membrane deforming or membrane sensing proteins (Farsad and De Camilli, 2003; McMahon and Gallop, 2005; Shibata et al., 2009). Specific membrane regions with a particular arch require a highly dynamical exchange between lipids and proteins to sense, generate and/or stabilize membrane curvature. To respect thermodynamic laws, very high membrane curvature is limited to short period of time or has to be stabilized by surrounding proteins. The elastic properties of lipid bilayers allow cellular membrane to resist to spontaneous bending and high curvature. Specific proteins domains or motifs that sense, stabilize or generate membrane curvature will deliver support and forces that levy tension on membranes while other proteins act directly on the membrane by changing lipids (McMahon and Boucrot, 2015). Membrane-bending mechanisms are based on three parameters (Johannes et al., 2014): chirality (asymmetry of lipid and protein material of the membrane), scaffolding (rigid and arched proteins such BAR-domain that induce membrane deformation after membrane binding; Frost et al., 2009; Mim and Unger, 2012) and crowding (concentration of proteins on a specific membrane domain inducing deformation; Stachowiak et al., 2010, 2012). It has been shown that vesicle budding process requires the presence of different coat proteins (such as Clathrin and COPI/II) to stabilize membrane curvature; Kirchhaussen, 2000; McMahon and Boucrot, 2011). Coat proteins do not interact directly with lipids and count on adaptor proteins (such AP2) to link them to membranes. Caveolar vesicles are formed by oligomerization of caveolin with a hairpin loop inserted within the membrane thus enabling membrane deformation and stabilization (Parton and Del Pozo, 2013). Other proteins, such as dynamin, are able to polymerize into spirals and induce membrane deformation (Ferguson and De Camilli, 2012), but such process will require an pre-existing curvature (Morlot and Roux, 2013). On the other hand, lipid nature is a key in initiating membrane deformation process. One example is the initiation of clathrin-mediated endocytosis where AP2 and clathrin recruitment to the membrane require PI(4,5)P_2_ lipid to initiate the process (Cocucci et al., 2012). Another example highlighted how the presence of polyunsaturated phospholipids increase the deformation and vesiculation of synthetic membrane by dynamin and endophilin (Pinot et al., 2014). This is an important mechanism in synaptic vesicles, where phospholipids with polyunsaturated acyl chains are extremely abundant.

*In vitro* studies using membrane model can interrogate the curvature effect on membrane–protein interactions. An example presented here shows that lipid composition is not the only driver for DYS R11-15 to interact with membrane, but also the curvature. Increasing liposome concentration in the presence of DYS R11-15 enabled steady-state tryptophan fluorescence (Figure 3C). Increasing the concentration of anionic SUV increase tryptophan fluorescence of DYS R11-15 until it reached a plateau. On the contrary, anionic LUVs with different size decreased tryptophan fluorescence (Figure 3C) and similar observation were obtained with zwitterionic SUVs and LUVs (Sarkis et al., 2011). In the case of DOPC: DOPE SUVs, no significant response is obtained by Biacore, whereas a decrease in tryptophan fluorescence was observed when the partners are together in solution. The DYS R11-15-DOPC: DOPE SUVs interaction therefore appears weak and undetectable by Biacore. This method requires the existence of a continuous flow in the microfluidic system. This flow may be sufficient to break low interactions, which is likely the case here. On the other hand, the dissociation kinetics of DYS R 11-15 in the presence of DOPC: DOPS SUVs are slow and the signal did not reach base level. The binding of the protein on these anionic vesicles is therefore strong and not completely broken by the microfluidic flow. We observed by fluorescence measurement that the tryptophan residues of the protein are exposed to a much more hydrophobic environment in the presence of anionic than zwitterionic SUVs, and concluded that the interaction of DYS R11-15 with SUVs is considerably stronger with anionic lipids as with zwitterionic lipids (Sarkis et al., 2011). The analyzes by Biacore thus confirm this conclusion.

The accessibility of Lysine and arginine in DYS R11-15 alone or when in contact with SUVs was further investigated. LC/MS/MS of trypsinized peptides alone or in the presence of lipid vesicles allowed the identification of a protein protected zone from trypsin action when in contact with anionic DOPC/DOPS vesicles (Figure 3D), validating the changes observed in tryptophan fluorescence. Interestingly, similar results are not observed when the protein was in contact with zwitterionic DOPC/DOPE SUVs, validating the importance of lipid nature and charge for an efficient binding. Overall, these data are in a good agreement with previous observations, demonstrating that dystrophin fragments undergo structural rearrangement when binding to membranes (Legardinier et al., 2009).

Lipid vesicles of various size with single lipid species can undergo mechanical forces thus adopting different shapes. Some insights on how the dynamic of GUV adsorption and deformation/rupture mechanism onto silicone oil-water interfaces and modified glass surface was discussed recently (Kataoka-Hamai and Kawakami, 2019). As GUV size allows direct visualization under fluorescent microscope, it is a popular membrane model to directly investigate protein binding or to impose mechanical force thus deforming the membrane. A microscopy setup that combines a micropipette and optical tweezers are frequently used to manipulate GUV and impose membrane deformation. GUV membrane tension is controlled using the micropipette whereas optical tweezers is used to pull out the membrane and form a tube while measuring the required force to control the tube. Using such system allowed to investigate the force required through dynamin polymerization to efficiently deform membranes, and showed that the polymer nucleation of dynamin depends on the membrane curvature (Roux et al., 2010). Moreover, such model is used to isolate the effect of membrane shape, for instance, the KvAP (voltage-gated ion channel protein) was enriched in curved membrane while AQP0 (major intrinsic protein of lens fiber) was indistinguishably present in flat and curved membrane (Aimon et al., 2014). Microaspiration (micropipette) provides a useful tool to measure not only reconstituted bilayer model but also cell membrane stiffness (Oh et al., 2012). Using this technique, we can assess global measurements of membrane deformability, unlike AFM, which gives more information on local bilayer stiffness (Okajima, 2012). Nevertheless, tethering the membrane of a cell into a nanotube using optical tweezers can be used to better understand rheological and biophysical properties generated by the interaction of a cell bilayer with the cytoskeleton. Tethering cellular membrane can also be achieved using AFM (Ou-Yang and Wei, 2010). GUV reconstitution with membrane proteins were also widely used to investigate functional transport or protein translocation (Kedrov et al., 2013).

Phosphoinositides (PI) phospholipid family was shown to be involved in many process such as membrane dynamic and signal transduction (Di Paolo and De Camilli, 2006). The binding with stereospecific protein recognition containing modular domains to distinct headgroup of PI has been widely studied, especially with Pleckstrin homology (PH) domain, FYVE domain and PX domain (Moravcevic et al., 2012). More recently, DrrA protein involved in Lung macrophage infection by *Legionella pneumophila*, has been showed to binds specifically to phosphatidylinositol 4-phosphate (PI4P) lipid.

Crystal structure of DrrA with PI4P and isothermal titration calorimetry experiments revealed selectivity and unexpectedly high affinity between the protein and the lipid (Schoebel et al., 2010). Interestingly, using monolayer model, a biphasic surface pressure responses of DrrA was observed, due to the robust insertion of an hydrophobic helix of the protein into the acyl chain of PI(4)P phospholipid present in the film (Del Campo et al., 2014). We used GUV formed by gentle hydration as described by Rodriguez et al. (2005), where a Chloroform/Methanol mix of 20%DOPC, 15%DOPS, 35%DOPE, 30% Cholesterol, 0.1% DiIC18(3) with 1% PI4P or PI(3,4)P_2_ was dried under Argon flow then rehydrated with sucrose solution. As shown in Figure 3E, DrrA labeled with Alexa Fluor 647 via sortase showed a strong and specific binding to GUV containing 1% PI4P but such strong binding was lost when switching the 1% PI4P to 1% PI(3,4)P_2_.

### Supported Lipid Bilayers: Lipid and Protein Diffusion

Supported lipid bilayer on a solid substrate is another biomimetic model that have found widespread use to mimic cell membrane and to study the physicochemical properties of lipids and the interaction with proteins (Sackmann, 1996; Milhiet et al., 2002). Only in recent years, structural details on SLB formation started to develop from both experimental (Keller and Kasemo, 1998; Jass et al., 2000; Reviakine and Brisson, 2000) as well as theoretical (Seifert, 1997; Zhdanov et al., 1999) investigations, but details on the forces required to drive the SLB process is still unclear (Richter et al., 2006).

Usually, the bilayer is adsorbed on a solid hydrophilic substrate by vesicle fusion or using a layer by layer deposition (Langmuir-Blodget and Langmuir-Schaefer [LB + LS]), thus allowing quantitative experiments with local techniques such as AFM (Richter and Brisson, 2005), fluorescence correlation spectroscopy (Zhang and Granick, 2005a), fluorescence recovery (Tamm and McConnell, 1985), and others (Seantier et al., 2004; Daillant et al., 2005). Such variety of techniques allowed to access at the nanometer scale, spatiotemporal information and dynamical organization of SLB formation, all emphasizing the role of electrostatic interactions in the process. The nature of the used substrate to form SLB control the membrane properties, and understanding that is important before using the membrane model for further investigation (Tanaka and Sackmann, 2005). Most substrates used to form SLB are mica and glass, and many studies identified the role of bivalent cations such as calcium as well as the vesicle size effect on rupture (Reviakine and Brisson, 2000; Reimhult et al., 2002) but other details remain vague. Another important parameter to be considered is the bilayer fluidity related to the lipid lateral mobility, and this might be influenced not only by lipid structure/nature (Wagner and Tamm, 2000), but also lipid labeling (Tamm, 1988), substrate nature and cleaning procedure (Seu et al., 2007). The interaction between the membrane proximal leaflet and the substrate is thought to affect lipid diffusion in the monolayer and could be different than the distal leaflet (Tamm, 1988; Zhang and Granick, 2005b; Xing and Faller, 2008), thus affecting the overall bilayer fluidity (Schmitt et al., 2001; Przybylo et al., 2006). Lipid bilayers are separated by a thin water gap from the substrate and the thickness of that water layer depends on the material of the substrate (∼1 nm when using hydrophilic glass). Many researches aimed to suspend the bilayer on polymer or other cushion to avoid such direct interaction or increase that thickness by engineering a floating bilayer membrane (Su et al., 2020). The first study investigating the lipid diffusion (Tamm and McConnell, 1985) in a single SLB was performed using a silicon substrate.

**FIGURE 4:**
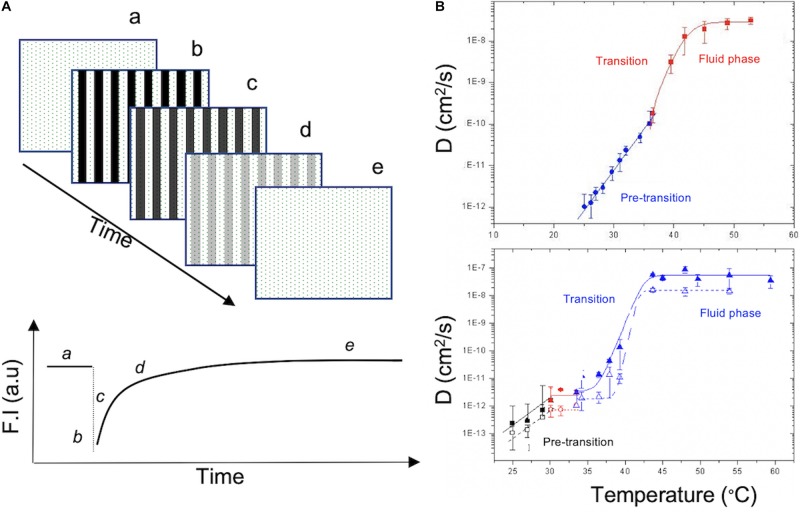
**(A)** Fluorescence Recovery After Patterned Photobleaching (FRAPP) used to probe phospholipid diffusion in SLB. Fluorescent SLB **(a)** was photobleached in fringe pattern and the overall intensity decrease **(b)**. Over time, lipids diffuse, and a recovery of the fluorescence is observed **(c–e)**. Modified from Harb et al. (2012). **(B)** Diffusion coefficient of DPPC membrane supported on glass (top) or mica (bottom) measured using FRAPP while varying the temperature of the SLB from 20 to 55°C. In the bottom graph, full symbols correspond to the distal leaflet and empty symbols correspond to the proximal leaflet due to its interaction with the mica substrate. Slower diffusion is observed at low temperature and it increases with higher temperature until it reaches a plateau during the fluid phase. Reproduced by J. S. with permission (Scomparin et al., 2009).

We investigated in detailed the same setup and under identical conditions, the formation of DPPC (1,2-dipalmitoyl-sn-glycero-3-phosphocholine) SLB using LB + LS deposition on two different substrates, mica and glass, and investigated lipid diffusion in these bilayer using fluorescence recovery after patterned photobleaching (FRAPP) (Davoust et al., 1982; Nkodo and Tinland, 2002; Jourdainne et al., 2008) (Figure 4A). Similar design was used to investigate the insertion of α-hemolysin protein into SLB and measuring the pore complex diffusing freely in the membrane (Harb et al., 2012). Other studies generated suspended lipid bilayers (on microstructured Si/SiO2 chips or on polymer brush; Kusters et al., 2014; Mashaghi and van Oijen, 2014) used to investigate membrane transport or viral fusion (Kusters et al., 2014). Even more complex *in vitro* model consists of using receptor and ligand pair reconstituted in two different membrane model GUV and SLB (Carbone et al., 2017).

Regarding the FRAPP measurement, briefly, the light beam of an Ar laser was split into two equivalent fringe patterns (Davoust et al., 1982; Scomparin et al., 2009; Harb et al., 2012). Labeled bilayer was photobleached by increasing the laser to full intensity for a short period of time (less then 1 s) in the illuminated fringes. An optical fiber and a photomultiplier will measure the decrease of the intensity of the bilayer after photobleaching as well as the recovery over time (Figure 4A). Using the fringe patterns, small diffusion deviations can be detected thus being a perfect tool to investigate substrate effect and to decouple the dynamics of the two leaflets in the bilayer.

DPPS bilayer [containing 0.1% of 16:0-12:0 NBD PC (nitrobenzoxadiazole)] obtained by Langmuir-Blodget followed by Langmuir-Schaefer (LB + LS) transfer (Girard-Egrot and Blum, 2007) resulted in a homogeneous large domain. Increasing temperature drive a gel to fluid transition and consequently increase the diffusion coefficient (Figure 4B). DPPC Bilayer interaction with glass substrate appears to be low as FRAPP signals showed a mono-exponential fluorescence decay indicating a uniform dynamic in both leaflets of the membrane (Figure 4B, top graph). On the other hand, using mica showed a biphasic response to photobleaching (Bi-exponential signal) suggesting dynamical differences between proximal versus distal leaflet, even though transition temperature is alike (Figure 4B, bottom graph). Stronger interaction between membrane and substrate is observed with mica due to its higher zeta potential and its surface tropology (atomically flat) compared to glass substrate, hence a slower diffusion in the proximal than in the distal lipid leaflet. In comparable conditions, vesicle fusion was used to form SLB leading to more heterogenous behaviors with similar effect of the solid substrate when using DMPC (1,2-dimyristoyl-sn-glycero-3-phosphocholine) bilayer (Scomparin et al., 2009).

The combination of membrane models and single-molecule fluorescence microscopy became an exciting research area to investigate binding and diffusion events of individual molecules (Weiss, 1999; Michalet et al., 2003; Mashaghi and van Oijen, 2014; Cheney et al., 2017). Novel optics and cameras with high signal to noise ratio made single molecule detection possible under low molecule concentrations (Gell et al., 2006). These conditions are optimal when using total internal reflection fluorescence microscopy (TIRF), where an evanescent field exponentially decaying near the coverslip is generated after the incident laser beam being totally reflected at the interface (glass-water). The great benefit of such system is the detection of signal from molecules present only in the evanescence field and within 100 nm from the glass surface, while the rest of the molecules in solution above cannot be spotted (Gell et al., 2006). With such high sensitivity, some experimental conditions should be respected, especially related to the concentration of the fluorescent analytes which should not pass couple of nM range. This is crucial to make sure that the identified intensity in one diffraction-limited pixel/volume is coming from one molecule only (van Oijen, 2011). This powerful technique has been used on live cells to follow the dynamic of proteins interacting with cellular membrane, as well as on membrane models such as SLB (Mashanov and Molloy, 2007; Fox et al., 2009; Cocucci et al., 2012; Rozovsky et al., 2012). Biomolecular interactions are keys to execute biological tasks (signaling, activation, inhibition…) and measuring binding has been widely studied using many different assays. However, single molecule methods allow, not only to assess binding rates with high precision, but more importantly to observe heterogeneity in binding characteristics.

**FIGURE 5:**
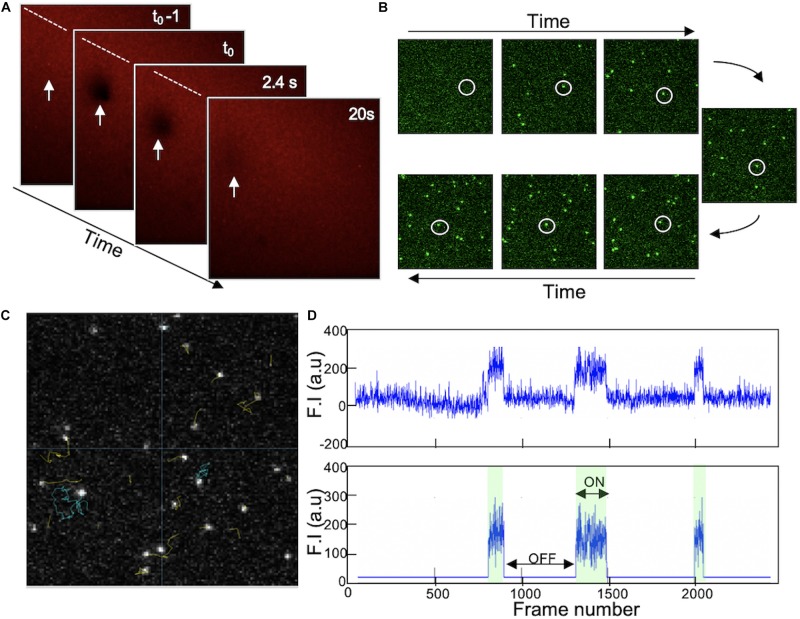
**(A)** Supported lipid bilayer on glass formed by vesicle fusion of SUVs (78.9% DOPC, 20% DOPS, and 1% PI4P) in the presence of CaCl_2_. Homogeneous fluorescent signal is detected due to 0.1% DiIC18(3) added to the lipid mix. Fluorescence photobleaching (white arrow) was performed using a confocal microscope and the recovery of the intensity validate the lateral lipid diffusion and the quality of the SLB. Photobleaching occurs at t_0_. **(B)** TIRF images acquired at different timepoint when injecting DrrA on SLB. Fluorescently labeled protein (DrrA sortase labeled with Alexa Fluor 647) was added at 100 pM on the top of SLB containing 1% PI4P. The interaction of DrrA with membrane was observed using TIRF microscopy allowing higher resolution for single molecule tracking. The white circles showed the arrival of DrrA molecule after injection and its diffusion on the surface of SLB. Images are 128 × 128 pixels. **(C)** Processed image shows trajectory of individual protein diffusing on the membrane analyzed using MatLab. **(D)** Fluorescence traces over time of individual protein (raw signal on top, and background processed signal at the bottom) showing the protein residency time (Binding or ON events) and the unbinding (OFF events). All reproduced by J. S. from Schoebel et al. (2010); Del Campo et al. (2014), and Sidm and Wang (2016).

Using a microfluidic chip, SUVs containing 78.9% DOPC, 20% DOPS, 1% PI4P, and 0.1% DiIC18(3) were incubated on a freshly cleaned and plasma treated glass slide in the presence of calcium. Observing the SLB formation under the confocal microscope shows the biding of the fluorescent SUV to the glass and bursting into bilayer until a homogeneous signal was obtain after 20 min. Moreover, the quality of the formed SLB was validated by FRAP and a fast recovery of the fluorescence was observed, indicating a homogeneous bilayer and the fluid state of the membrane (Figure 5A). DrrA protein labeled with Alexa Fluor 647 by Sortase injected on the surface of the SLB at very low concentration (100pM). Similarly to GUV models, only the presence of 1% of PI4P in the membrane allows DrrA protein to bind and diffuse freely in 2 dimensions on the bilayer as shown in Figure 5B. It was important to have low number of proteins (very low concentration) to avoid having multiple protein in one pixel and to decrease the collision between neighbor molecules diffusing on the membrane thus making the single molecule tracking more difficult. Each individual protein trajectory was detected and tracked throughout its diffusion on the membrane (Figure 5C) and traces showing the residency time of individual DrrA when interacting and moving on the SLB can be quantified (Figure 5D, top trace). The fluorescence intensity for each object was corrected for background fluorescence (Figure 5D, bottom trace), and fitting all ‘on events’ histogram allows residency time quantification of the protein on the membrane, hence its off rate.

Combining single molecule imaging and SLB is the appropriate strategy to directly visualize binding kinetics between proteins, peptides, nucleic acid, viral particles, etc. and membrane. This is getting more interest from the scientific community particularly to visualize proteins/molecules that have low binding affinity to membrane when conditions are optimized (fast camera chip and fluorophore stabilizing reagent).

## Conclusion

To conclude, it is worth noting that this review is not exhaustive and might not present the last advances on biomimetic membranes, but it certainly provides an overview on main membrane models used for lipid–protein interaction studies. In many cases, multidisciplinary approaches are required to tease apart obstacles and to understand biological process affected by physico-chemical and mechanical properties. Ultimately, progress using such models will continue to bring to the field better understanding on biomolecular interactions and membrane dynamics and help tackles biomedical problems related to membrane-pathogenicity.

## Author Contributions

JS and VV contributed to the final manuscript.

## Conflict of Interest

The authors declare that the research was conducted in the absence of any commercial or financial relationships that could be construed as a potential conflict of interest.
